# Supplementary material for: Descriptive analysis of surveillance data for Zika virus disease and Zika virus-associated neurological complications in Colombia, 2015–2017
Source: PLoS One. 2021 Jun 16;16(6):e0252236. doi: 10.1371/journal.pone.0252236 (PMC8208586; doi:10.1371/journal.pone.0252236)
Supplement: Attachment — Submitted filename: Peer Review Response Letter.docx [file pone.0252236.s001.docx]

MRC Centre for Global Infectious Disease Analysis
Department of Infectious Disease Epidemiology
Imperial College London

St Mary’s Campus, Norfolk Place
London W2 1PG
United Kingdom

k.charniga17@imperial.ac.uk

April 21, 2021	**Kelly Charniga BSc MPH**

Research Postgraduate

Berlin Londoño, PhD

Joerg Heber, PhD

Editors

*PLOS ONE*

Dear Dr. Londoño, Dr. Heber, and Reviewer 1,

Thank you for sending the peer review comments on our manuscript entitled “**Descriptive analysis of surveillance data for Zika virus disease and Zika virus-associated neurological complications in Colombia, 2015-2017**.” We greatly appreciate the insights gained through the peer review process and have carefully considered each comment. We believe the paper is stronger as a result and hope you find the changes satisfactory.

In this letter, the line numbers from the peer review comments correspond to the first version of the paper, whereas the line numbers in our response correspond to the new version.

Regarding data availability, we have revised our statement in lines 432-440:

The neurological complications dataset cannot be shared publicly as the data contain information on a small number of patients. Although the data are anonymized, identification is a risk given the high geographic resolution and large combination of predictor variables. This determination was made by Comité de Ética y Metodologías de Investigación (CEMIN). To request access to these data, please contact: [secretariactin-cein@ins.gov.co](mailto:secretariactin-cein@ins.gov.co), (57+1) 2207700 Ext. 1331-1108, Colombia. The aggregated ZIKV dataset is available on GitHub: https://github.com/kcharniga/descriptive_zika. The data include the number of weekly reported cases by administrative level 2 (municipality) as well as sex and age category.

The GitHub repository is currently set to private mode. If the paper is accepted for publication, the GitHub repository will be made public.

Regarding Figures 2 and 6, the maps were originally created using GADM version 2. However, we have now replaced them with maps created using shapefiles from the Humanitarian Data Exchange (<https://data.humdata.org>). These data are available under a Creative Commons Attribution for Intergovernmental Organizations (CC BY-IGO) license (<https://data.humdata.org/about/license>), which is compatible with CC BY 4.0. The figure legends have been updated accordingly.

In response to Reviewer 1:

Comment 1: The authors should describe the evidence for Zika virus (ZKV) being neurotrophic. Evidence of ZKV neurotropism is not only found in patients with Guillain-Barré syndrome (GBS) or congenital zika virus syndrome (CZS), but there is preclinical evidence from animal studies showing that zika virus can infect the central nervous system in both young and adult animals (rodents and nonhuman primates). Explaining the evidence for neurotropism can help to explain why more neurologic symptoms beyond GBS should be investigated after ZKV infection in patients (Discussion pg 16 ln 371-379).

We fully agree with this comment and have added additional information about the neurotropism of ZIKV to lines 390-395.

Comment 2: According to the current study data the highest incidents of ZVD is shown in adults. However, the data also shows that children of both sexes are equally susceptible to ZVD. In fact, a recent prospective study of children in Columbia (Pacheco et al, 2021, Paediatr Perinat Epidemiol, 35: 92-97) demonstrates that 15% of children infected with Zika virus between 1-12 month of age had adverse neurologic, hearing or eye examinations at 20 - 30 months of age. An additional 12.8% received an alert score in the hearing domain. Considering the neurotropism of the virus and the considerable postnatal development from birth to young adulthood, it is important to include broader surveillance of this young population other than severe neurological complications (see also recent review Raper & Chahroudi, 2021, Trop Med Infect Dis, 6 (10): 1-12).

We would like to thank the reviewer for suggesting these references. In lines 94-97, we have added additional information about postnatal transmission of ZIKV. We have also added the findings from the Pacheco et al. study to lines 417-420.

Comment 3: Discussion pg 14 Ln 331-334, the authors should briefly mention that the possibility of prior immunity is also not possible due to the timing and origins of the virus arriving in Latin America from French-Polynesia.

We thank the reviewer for this comment and have made the adjustment to lines 338-339.

Comment 4: Discussion pg 14 Ln 344-346, if spending more time at home is an increased risk of mosquito bite and ZVD in older females, would this also be the case for young children?

The reviewer makes a valid point. We agree, and have expanded our discussion of household-based exposure to mosquito bites in lines 356-359.

Thank you again for helping us to improve our manuscript.

Sincerely,

Kelly Charniga
